# Confined B‐Cell Reconstruction and High T‐Cell Clonality Define Clinical Response to Cladribine Treatment

**DOI:** 10.1002/ana.78165

**Published:** 2026-01-23

**Authors:** Tilman Schneider‐Hohendorf, Maria Eveslage, Timo Wirth, Christian Wünsch, Eva M. Schumann, Jan D. Lünemann, Heinz Wiendl, Luisa Klotz, Nicholas Schwab

**Affiliations:** ^1^ Department of Neurology University Hospital Münster Münster Germany; ^2^ Institute of Biostatistics and Clinical Research University of Münster Münster Germany; ^3^ Department of Neurology and Neurophysiology, Medical Center University of Freiburg Freiburg Germany

## Abstract

Cladribine tablets are approved for relapsing multiple sclerosis, mediating their clinical effect by moderately depleting lymphocytes. In a prospective, monocentric study including 22 patients completing 2 annual cycles of cladribine, B‐ and T‐cell receptor repertoires and relapse activity were assessed at baseline and after 24 months. T‐cell clonality increased, driven by loss of low‐frequency, naive clonotypes, and re‐expansion of dominant CD8 memory clonotypes, particularly in clinically stable patients. In contrast, B‐cell receptor richness increased because of reconstruction by transitional and naive B cells with higher clonotype numbers observed in relapsing patients. Therefore, competing immune reconstitution following cladribine therapy could result in differential clinical responses. ANN NEUROL 2026;99:1166–1172

Multiple sclerosis (MS) is a chronic, immune‐mediated disease, characterized by central nervous system (CNS) infiltration of immune cells, cortical demyelination, and progressive neuroaxonal loss.^1^ Although the precise pathophysiology of MS remains incompletely understood, the efficacy of systemic immunomodulatory and immunodepleting therapies in reducing relapse rates supports a key role for CNS infiltrating T and B lymphocytes in lesion formation and disease progression.^2^ Approved for the treatment of highly active relapsing MS, cladribine is an oral therapy in the form of 2‐chlorodeoxyadenosine, mimicking the nucleoside deoxyadenosine, thereby interfering with DNA processing in dividing cells. Originally developed for hematologic malignancies, cladribine preferentially accumulates in lymphocytes, leading to apoptosis and transient lymphocytopenia.^3^ It is applied in 2 annual treatment cycles, but shows sustained clinical effects beyond the 2 treatment years.^4^ B‐cell depletion and repopulation are more profound after both treatment cycles compared to T cells, which exhibit less profound reduction and incomplete repopulation after 2 years relative to baseline levels.^5^

This clinical trial assessed the effects of 2 completed, approved cladribine cycles on peripheral B‐ and T‐cell receptor clonality and richness, validated by flow cytometry, and investigated their potential links to recurrent disease activity.

## Methods

### Study Cohort and Ethics

The study cohort comprised 24 relapsing–remitting MS (RRMS) patients recruited for a monocentric, open‐labeled, explorative, prospective, single‐arm study over 24 months and 2 consecutive and completed cladribine treatment cycles (ClaiMS study, EU Clinical Trials Register EudraCT no.: 2018–004557‐24), investigating the mechanism of action of cladribine therapy with the end‐points defined as changes in T‐cell and B‐cell receptor repertoire clonality, unique immune receptor clonotypes (richness), and templates (sequenced cells). The study was conducted in accordance with the Declaration of Helsinki and approved by the local ethics committee of the Ärztekammer Westfalen‐Lippe (2019‐391‐f‐A). All participants provided written informed consent before enrollment. Number of relapses and Expanded Disability Status Scale were assessed at baseline and over a follow‐up of 24 months after the start of the treatment. Eleven participants relapsed during the study, 9 of them relapsed within the first 12 months of cladribine therapy. From 2 patients, biomaterial was unavailable for immune receptor sequencing (Table 1).

**TABLE 1:** Demographics and Clinical Characteristics of the Study Cohort

RRMS patient n	F/M	Mean age	Disease duration from symptom onset (yr)	Mean EDSS at baseline	Mean EDSS at month‐24	Mean no. of relapses at baseline	Relapsing patient n (relapse count)
22	14/8	35.6	7.74	1.55	1.45	3.62	11: 6 (1)/5 (2)

EDSS, Expanded Disability Status Scale; RRMS, relapsing–remitting multiple sclerosis.

### Bulk Immune Receptor Sequencing and Analysis

B‐cell receptor immunoglobulin heavy chain (IGH) and T‐cell receptor variable beta chain (TRB) sequencing was conducted from genomic DNA (gDNA) extracted from 10 million cryopreserved peripheral blood mononuclear cell (PBMC) using the Immunoseq assay (Adaptive Biotechnologies, Seattle/WA), including a baseline time point for TRB (n = 20) and IGH (n = 22) sequencing, a 12‐month time point for TRB sequencing (n = 18), and a 24‐month time point both for TRB (n = 19) and IGH (n = 22) sequencing. Repertoire data was analyzed as previously described,^6^ using the Immcantation framework for IGH.^7^ All changes were quantified as absolute changes. Quantification of virus‐specific T cells was performed as previously described.^8^ Briefly, HLA backgrounds of TRB repertoires were inferred using published HLA‐specific TRB sequences.^9^ Subsequently, strictly multimer‐derived TRB sequences were extracted from the VDJdb in July 2025 together with their HLA restriction, resulting in 894 CMV‐, 545 EBV‐, 497 influenza A‐, and 486 SARS‐CoV‐2‐specific TRB sequences of high confidence (Table S1). Extracted sequences were matched to TRB repertoires in an HLA background‐specific manner using TRB V gene and CDR3 amino acid sequence.

### Flow Cytometry Staining and Analysis

Flow cytometry staining was performed from 10 million cryopreserved PBMC as previously described (panels 2, 4 and 6),^10^ from 18 baseline and 20 month‐24 follow up samples (16 pairs). Three multicolor panels were assessed: a B‐cell subset panel (anti‐CD19, ‐CD20, ‐CD21, ‐CD23, ‐CD24, ‐CD27, ‐CD38, ‐CD80, ‐IgD, and ‐IgM); a T‐cell memory subset panel (anti‐CD3, ‐CD4, ‐CD8, ‐CD27, ‐CD28, ‐CD31, ‐CD45RA, ‐CD45RO, ‐CD56, ‐CD57, ‐CD62L, ‐CD197, ‐CD226), and a T‐helper cell (Th) subset panel (anti‐CD3, ‐CD4, ‐CD8, ‐CD27, ‐CD45RO, ‐CD56, ‐CD146, ‐CD183, ‐CD185, ‐CD194, ‐CD196, ‐ICOS, and ‐PD‐1). B‐cell subsets were defined as CD19+ CD20+, naive (IgD+, CD27−, CD38−), transitional (IgD+, CD27−, CD38+, CD24+), double‐negative (IgD−, CD27−), class‐switched memory (IgD−, CD27+), unswitched memory (IgD+, CD27+, CD21−), and marginal zone‐like B cells (IgD+, CD27+, CD21+). T‐cell memory subsets were defined as CD3+, CD4+, CD56−, or CD3+, CD8+, CD56−, naive (CD45RA+, CD62L−), central‐memory (CD45RA−, CD62L+), effector‐memory (CD45RA−, CD62L−), terminally differentiated effector‐memory (CD45RA+, CD62L−), and CD57+ for further discrimination of CD8 T‐cell subsets. Th subsets were defined as CD3+, CD4+, CD56−, CD45RO+, Th1 (CD194−, CD196−, CD183+), Th2 (CD194+, CD196−, CD183−), Th17 (CD194+, CD196+, CD183−), Th17.1 (CD194−, CD196+, CD183+, CD185−), and follicular Th cells (CD185+). Analysis was performed with Kaluza Analysis V2.3 (Beckman Coulter Brea/CA). T‐cell frequencies were calculated of CD3+ T cells, B‐cell frequencies of CD19+ CD20+ B cells. Changes were calculated as absolute changes.

## Results

### Differential Effects of Cladribine‐Mediated Lymphocyte Reduction on T‐Cell and B‐Cell Receptor Repertoires

**FIGURE 1:**
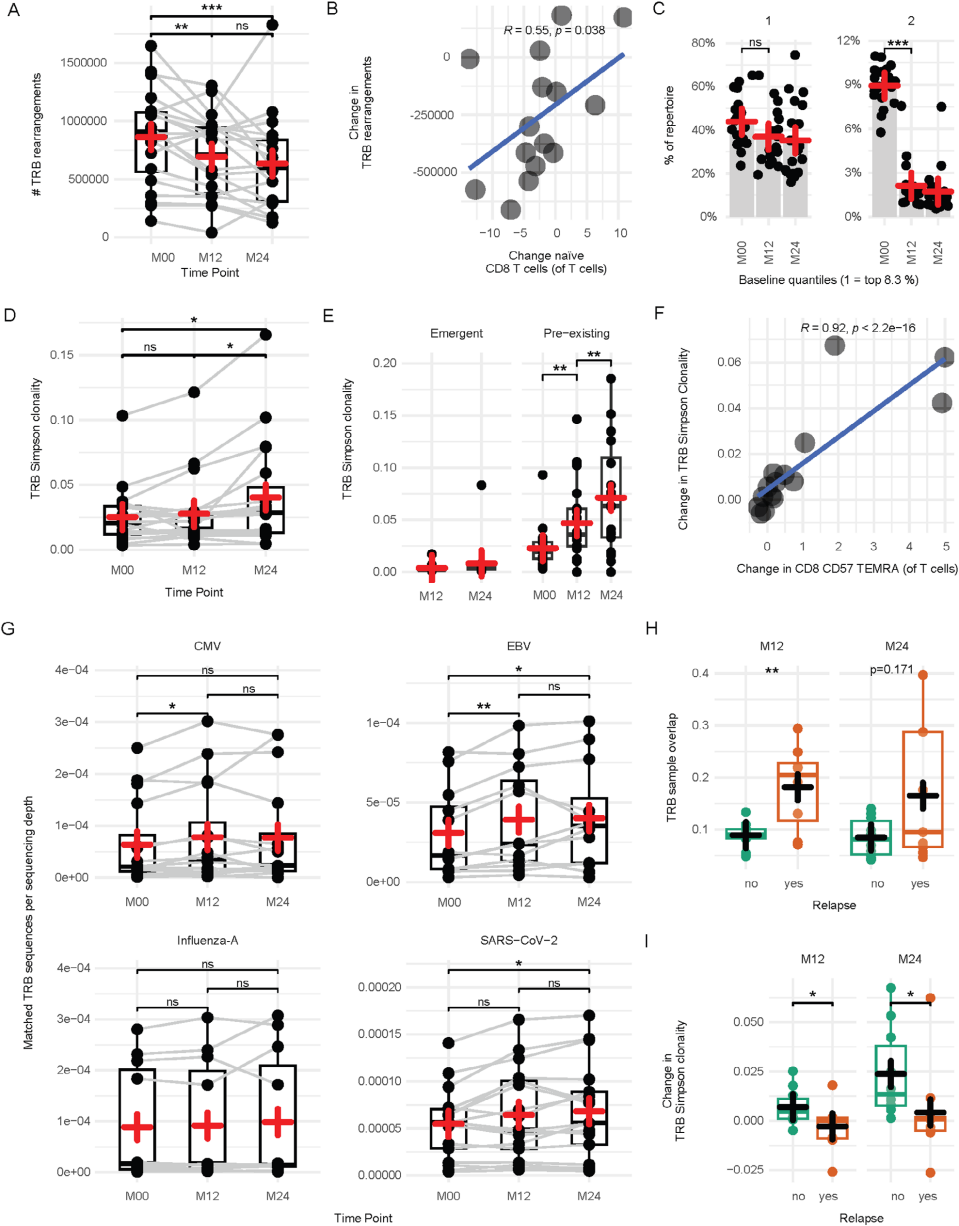
Cladribine‐mediated T‐cell depletion is associated with increased clonality and loss of low expanded T‐cell receptor variable beta (TRB) clonotypes. (A) Number of unique nucleotide T‐cell receptor beta chain  rearrangements. (B) Flow cytometry‐derived changes in percentages of naive CD8 T cells correlated with changes in unique TRB rearrangements from baseline to 24‐month follow‐up. (C) Percent of baseline (M00) repertoire occupied by unique nucleotide TRB rearrangements divided into 12 quantiles (8.3% of unique TRB rearrangements per quantile) according to clonal rank. For month 12 and 24, only pre‐existing TRB clonotypes were quantified, identified by nucleotide overlap with the baseline sample. Quantiles 1 and 2 are shown, all 12 quantiles are depicted in Fig S2B. (D) TRB repertoire Simpson clonality. (E) TRB repertoire Simpson clonality, divided into unique nucleotide TRB clonotypes already detectable at baseline (pre‐existing) and not detectable at baseline (emergent). (F) Changes in TRB Simpson clonality correlated to flow cytometry‐derived change in percentages of CD57‐expressing CD8 terminally differentiated effector‐memory T cells (TEMRA) from baseline to 24‐month follow‐up. (G) Number of CMV‐ (upper left), EBV‐ (upper right), influenza‐A‐ (lower left), and SARS‐CoV‐2‐specific T cells per sequencing depth, defined by unique TRB nucleotide rearrangements. (H) Unique nucleotide TRB rearrangement sample overlap from baseline to month 12 and from baseline to month 24, divided into patients with (n = 8 at M12 and n = 9 at M24) and without (n = 10) relapse activity. (I) Change in TRB repertoire Simpson clonality, divided into patients with (n = 8 at M12 and n = 9 at M24) and without (n = 10) relapse activity. R indicates the Spearman coefficient. Boxes indicate the 25% and 75% percentile and median, whiskers indicate 1.5× interquartile range, plus indicates the mean. Green dots and boxes indicate patients without relapses, red boxes and dots indicate patients with relapses. Significance was assessed using a paired Wilcoxon rank sum test where possible or a Mann–Whitney *U* test. *p < 0.05, **p < 0.01, ***p < 0.001, ****p < 0.0001. [Color figure can be viewed at www.annalsofneurology.org]

Quantification of unique nucleotide TRB rearrangements as a measure of TRB repertoire richness revealed an overall decrease from a baseline mean of 862,026 to 633,434 after 24 months with most of the decrease happening from baseline to month 12 (Fig 1A). Flow cytometry‐based assessment of T‐cell subsets revealed that the decrease in TRB richness positively correlated with significant changes in naive CD8 T cells (see Fig 1B, Fig S1, and Table S1). A similar result was obtained for TRB templates (ie, sequenced T cells), declining from a baseline mean of 1,479,299 to 1,117,551 after 24 months (Fig S2A). To evaluate in detail, which clonotypes are depleted, unique nucleotide TRB rearrangements of the baseline repertoire were divided into 12 quantiles according to clonal rank, and pre‐existing TRB clonotypes were identified in 12‐month and 24‐month samples by quantifying nucleotide TRB rearrangements already detectable at baseline. The first quantile group of the baseline time point, containing the top‐expanded memory TRB clonotypes, contained 44% of sequenced templates. Although cladribine‐mediated lymphocyte reduction resulted in a decrease of unique TRB rearrangements by 27% after 24 months, TRB template proportion of pre‐existing, expanded clonotypes remained unchanged in the first quantile group at month 12 and month 24. In contrast, profound decreases of pre‐existing TRB clonotypes from baseline to follow‐up time points were observed in all other quantile groups (see Fig 1C and Fig S2B). TRB clonality increased from a baseline of 0.025 to 0.040 after 24 months, where clonal expansion was evident specifically from month 12 to month 24, and was mediated by pre‐existing TRB clonotypes, while newly emerging clonotypes were not clonally expanded at month 12 or month 24 (Fig 1D‐E). Correlation with flow cytometry‐based T‐cell phenotyping revealed that the increase in clonality was associated with expansion of CD8 terminally differentiated effector‐memory T cells (TEMRA), particularly CD57‐expressing TEMRA (see Figs 1F, Fig S2C, and Table S1). Of note, CD57‐expressing TEMRA were the only T‐cell population that expanded from baseline to month 24. The T‐cell response against common pathogens (CMV, influenza‐A, EBV, and SARS‐CoV‐2) remained stable or increased after completion of cladribine therapy (Fig 1G). Together, this indicates that TRB clonality increases by specific loss of low‐expanded TRB clonotypes and (re‐)expansion of pre‐existing, predominantly CD8 T‐cell clonotypes into the created immunological niche by lymphopenia‐induced proliferation.

Nine of 20 patients with available TRB sequencing data experienced relapses within 24 months alongside cladribine therapy. Although there was no difference in relapsing patients compared to non‐relapsing patients regarding the decrease of unique TRB sequences (Fig S2D), stable patients with MS showed a more pronounced decrease in pre‐existing TRB clonotypes from baseline to month 12 and a similar trend from baseline to month 24, which was assessed by TRB nucleotide rearrangement sample overlap (see Fig 1H). Stable patients with MS showed a stronger increase in clonality from baseline to both follow up time points (see Fig 1I), together indicating that early loss of certain low‐expanded TRB specificities accompanied by expansion of pre‐existing and pre‐expanded TRB clonotypes is associated with clinical stability alongside cladribine therapy.

**FIGURE 2:**
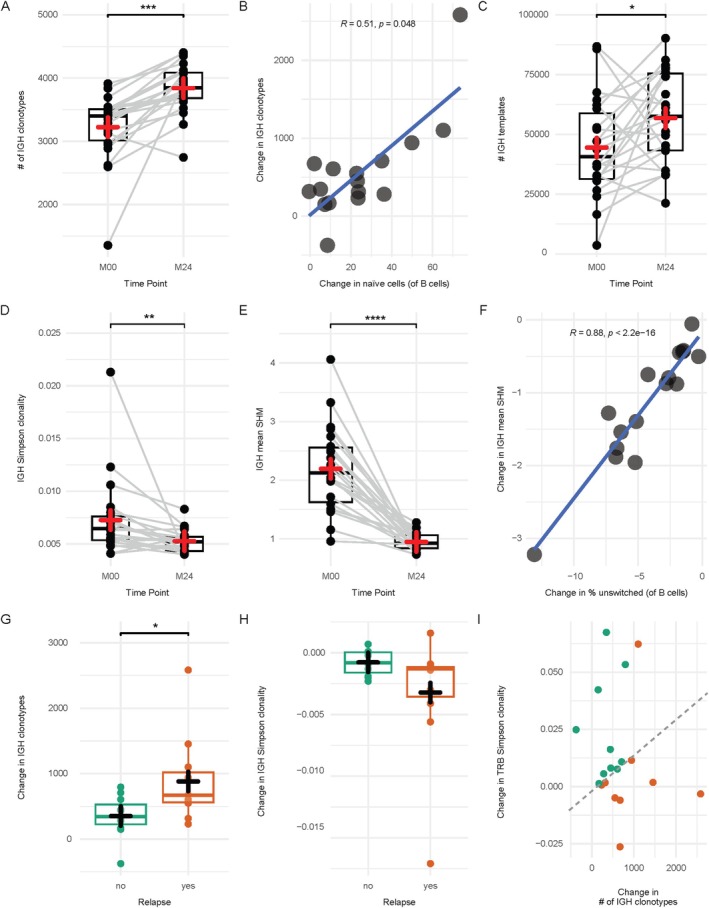
Cladribine‐mediated B‐cell depletion is associated with increased repertoire richness and reconstitution with naive and transitional B cells. (A) Number of phylogenetic tree immunoglobulin heavy chain (IGH) clonotypes. (B) Changes in flow cytometry‐derived percentages of naive B cells of B cells correlated with changes in IGH clonotypes from baseline to 24‐month follow‐up. (C) Number of IGH templates. (D) IGH repertoire Simpson clonality. (E) Mean somatic hypermutations (SHM) of IGH repertoires. (F) Changes in flow cytometry‐derived frequencies of unswitched memory B cells of B cells correlated with changes in IGH mean SHM from baseline to 24‐month follow‐up. (G) Changes in IGH clonotypes from baseline to month 24, divided into patients with (n = 11) and without (n = 11) relapse activity. (H) Changes in IGH repertoire Simpson clonality from baseline to month 24, divided into patients with (n = 11) and without (n = 11) relapse activity. (I) Correlation of data from G and Fig 1I (n = 9 relapsing patients vs n = 10 non‐relapsing patients). The manually drawn dashed line discriminates patients with and without relapse activity. R indicates the Spearman coefficient. Boxes indicate the 25% and 75% percentile and median, whiskers indicate 1.5× interquartile range, plus indicates the mean. Green dots and boxes indicate patients without relapses, red boxes and dots indicate patients with relapses. Significance was assessed using a paired Wilcoxon rank sum test where possible or a Mann–Whitney *U* test. *p < 0.05, **p < 0.01, ***p < 0.001, ****p < 0.0001. [Color figure can be viewed at www.annalsofneurology.org]

In clear contrast to cladribine‐mediated T‐cell reduction, which lead to a net loss of T‐cell specificities, cladribine‐mediated B‐cell depletion resulted in an increase of repertoire richness. IGH clonotypes, defined by specificity‐grouped phylogenetic trees, increased from a baseline mean of 3,224 to a 24‐month follow‐up mean of 3,841 (Fig 2A). Changes in IGH clonotypes positively correlated with flow cytometry‐assessed changes in percentages of naive B cells (see Fig 2B, Fig S3, and Table S1). IGH templates also increased from 42,846 to 55,449. This was accompanied by a decrease in IGH repertoire clonality from a value of 0.0073 to 0.0053, and a reduction of mean somatic hypermutations (SHM) from 2.19 to 0.95 (Fig 2C‐E). Flow cytometry‐based phenotyping confirmed depletion of all memory B‐cell subsets and increasing naive as well as transitional B‐cell frequencies (Fig S3). Changes in IGH mean SHM positively correlated with changes in frequencies of memory B‐cell subsets, most strongly with unswitched memory B cells (see Fig 2F), while naive B cells showed a negative correlation (Table S1). Together, this indicates profound depletion of the circulating memory B‐cell pool and reconstitution with transitional and naive B cells without relevant memory differentiation 12 months after the second cladribine cycle.

Relapse assessment revealed that stable patients with MS showed a less pronounced increase in IGH clonotypes (see Fig 2G) and a trend toward less pronounced decrease of IGH clonality, both after 24 months (see Fig 2H). When combining changes in TRB clonality and IGH clonotypes for a clinical assessment after 24 months, it became apparent that patients without relapses were characterized by a rise in TRB clonality, whereas patients with relapses were characterized by a rise in IGH clonotypes (see Fig 2I).

## Discussion

Using gDNA‐based immune receptor sequencing, we found that B‐cell depletion strongly affects the memory compartment, reflected in an overall decrease of SHM and IGH clonality. B‐cell repopulation is driven by reconstruction from the bone marrow, leading to a naive‐like B‐cell repertoire after year 2 with increased IGH richness, driven by higher transitional and naive B‐cell proportions. Using RNA‐based bulk sequencing of sorted B‐cell subsets, a previous study also reported decreases in memory B cells and increasing proportions of naive B cells concomitant with increased IGH diversity after year 1. Of note, this study additionally observed a tendency of reduced diversity within larger memory B‐cell clonotypes, indicating an increase of SHM in the small fraction of remaining memory B cells.^11^ A single‐cell RNA sequencing‐based study of peripheral blood and cerebrospinal fluid observed depletion of memory B‐cell subsets with high SHM levels, most strongly of switched memory B cells, as well as increased proportions of naive and transitional B cells with low SHM levels after year 2.^12^ Importantly, in the current study, a higher increase of naive‐like B‐cell clonotypes was associated with recurring disease activity. A similar association between counts of reconstituting B cells and recurring disease activity was also observed during ocrelizumab therapy,^13^ which depletes circulating CD20 B cells more durably and with differing reconstitution dynamics.^6^ In a prior flow cytometry‐based study, we suggested that part of cladribine's clinical effect could depend on the composition of the repopulated B‐cell repertoire 6 months after the first depletion cycle.^14^ Our data 12 months after completion of 2 cladribine cycles now indicate numerously reconstituting naive‐like B cells in relapsing patients, which could suggest an association between a high potential for B‐cell reconstitution /−differentiation and recurring disease activity.

Although cladribine‐mediated T‐cell reduction is less pronounced, it results in a loss of low‐expanded, naive‐like T‐cell clonotypes, which are not replenished after year 2. Second, it leads to increased repertoire clonality, which can in part be explained by preferential depletion of CD4 T cells,^5^, ^15^ which are less clonal compared to CD8 T cells. Specific interference of cladribine therapy with circulating CD4 Th cell subsets has been indicated before^15^, ^16^ and could affect B‐cell help with regard to establishment of putatively pathogenic meningeal B cells. Increased TRB clonality is in part mediated also by re‐expansion of previously expanded, mandatory clonotypes, most evident in CD57‐expressing CD8 TEMRA. These cells were previously shown to expand during immune reconstitution after alemtuzumab treatment^17^ and after hematopoietic stem cell transplantation,^18^ consistent with lymphopenia‐induced proliferation, and could include specificities against common viruses (CMV, EBV, influenza‐A, and SARS‐CoV‐2).^19^ The increase in anti‐EBV T cells clearly contrasts with ocrelizumab‐mediated reduction of the anti‐EBV response,^8^ indicating that cladribine does not deplete the EBV host repertoire (circulating B cells) as durably as ocrelizumab. Importantly, loss of T‐cell specificities and increased clonality were both associated with a beneficial treatment response in this study. This suggests that a reduction‐induced lack of T‐cell help to B cells could beneficially modulate the reconstructed B‐cell repertoire, thereby contributing to the clinical efficacy of cladribine.

This study has limitations. Bulk TRB and IGH sequencing were both performed using gDNA to exclude influence of cellular activation states and transcriptional variability and to provide a robust assessment of clonality, richness, and clonal abundance. However, bulk RNA sequencing would provide better resolution of SHM with approximation of affinity maturation, and cell sorting could assign the observed global repertoire changes to specific B‐cell subsets. Single‐cell RNA sequencing, although limited in sequencing depth, could provide information of both immune receptor chains together with the cellular phenotype. Second, this study specifically investigated the IGH and TRB repertoire 1 year after completion of 2 approved cycles of cladribine with special regard to clinical outcomes. However, immune reconstitution dynamics strongly differ for B and T cells after cladribine treatment, and immune reconstitution is not completed 1 year after the second cycle.

Combining the immunological observations with clinical data 1 year after the second cladribine cycle suggests that patients either repopulate the immunological niche created by cladribine by expanding prior existing, highly expanded T‐cell clonotypes or by newly generated, transitional and naive B cells. The latter group of patients experienced relapses during the study. Future studies with prolonged observation times and higher patient numbers are warranted to further delineate the phenotype of repopulating, putatively pathogenic memory B cells and low rank T‐cell clonotypes in association with recurring disease activity after cladribine treatment.

## Author Contributions

Conception and design of the study: L.K., N.S. Acquisition and analysis of data: T.S.H., M.E., T.W., C.W., E.M.S., V.T., N.S. Drafting a significant portion of the manuscript or figures: T.S.H., J.D.L., H.W., L.K., N.S.

## Potential Conflicts of Interest

L.K. received research funding by Merck Germany. All other authors report no conflicts of interest related to this study.

## Supporting information


**FIGURE S1:** Flow cytometry characterization of T‐cell memory and T‐helper subset frequencies. The plots depict percentages of CD4 and CD8 memory T‐cell subsets and CD4 T‐helper cell (Th) subsets, quantified of CD3+ CD56− lymphocytes at baseline (M00) and one year after the 2nd cladribine cycle (M24) (EM = effector‐memory cells, CM = central‐memory cells, TEMRA = terminally differentiated effector‐memory cells, TH = T‐helper cells, TFH = follicular T‐helper cells). Boxes indicate the 25% and 75% percentile and median, whiskers indicate 1.5× inter‐quartile range. *P* values were generated using a paired Wilcoxon rank sum test.
**FIGURE S2:** Changes in TRB parameters alongside cladribine therapy. (A) Number of TRB rearrangement templates. (B) Percent of baseline (M00) repertoire occupied by unique nucleotide TRB rearrangements divided into 12 quantiles (8.3% of unique TRB rearrangements per quantile) according to clonal rank. For month 12 and 24, only pre‐existing TRB clonotypes were quantified, identified by nucleotide overlap with the baseline sample. (C) Changes in TRB Simpson clonality correlated to flow cytometry‐derived change in percentages of CD8 terminally differentiated effector‐memory T cells (TEMRA) from baseline to 24‐month follow‐up. (D) Changes in unique nucleotide TRB rearrangements from baseline to month 12 and month 24, divided into patients with (n = 8 at M12 and n = 9 at M24) and without relapse activity (n = 10).
**FIGURE S3:** Flow cytometry characterization of B‐cell subset frequencies. The plots depict percentages of B‐cell subsets quantified of CD19+ CD20+ B cells at baseline (M00) and 1 year after the 2nd cladribine cycle (M24) (MZ‐like = marginal zone‐like). Boxes indicate the 25% and 75% percentile and median, whiskers indicate 1.5× inter‐quartile range. *P* values were generated using a paired Wilcoxon rank sum test.

**Table S1:** Correlations of immune receptor sequencing parameters with flow cytometry quantifications and list of antigen‐specific TRB sequences. Sheets 1–4 contain Spearman coefficients (column B) and p values (column C) from correlations of flow cytometry‐derived changes (column A: tcell, th1 and bcell indicate the flow cytometry panel) in immune cell subset frequencies with changes in numbers of TRB clonotypes/rearrangements (sheet 1: Tcell_corr_clonotypes), TRB clonality (sheet 2: Tcell_corr_clonality), numbers of IGH clonotypes (sheet 3: Bcell_corr_clonotypes), and IGH mean SHM (sheet 4: Bcell_corr_shm). Sheet 5 (TRB_sequences_list) contains all antigen‐specific TRB sequences extracted from public repositories and used for quantification of Fig 1I, including organism (column A: EBV, CMV, Influenza A and SARS‐CoV‐2), protein (column B), epitope (column C), HLA restriction (column D), TRB variable gene restriction (column E), TRB junctional gene restriction (column F), and the TRB CDR3 amino acid sequence (column G).

## Data Availability

Bulk TRB and IGH sequencing data are shared in a public repository, accessible with the following link: https://doi.org/10.5281/zenodo.18152565.
