# Supplementary material for: Confined B‐Cell Reconstruction and High T‐Cell Clonality Define Clinical Response to Cladribine Treatment
Source: Ann Neurol. 2026 Jan 23;99(5):1166–72. doi: 10.1002/ana.78165 (PMC13092775; doi:10.1002/ana.78165)
Supplement: Supplementary file 1 — FIGURE S1: Flow cytometry characterization of T‐cell memory and T‐helper subset frequencies. The plots depict percentages of CD4 and CD8 memory T‐cell subsets and CD4 T‐helper cell (Th) subsets, quantified of CD3+ CD56− lymphocytes at baseline (M00) and one year after the 2nd cladribine cycle (M24) (EM = effector‐memory cells, CM = central‐memory cells, TEMRA = terminally differentiated effector‐memory cells, TH = T‐helper cells, TFH = follicular T‐helper cells). Boxes indicate the 25% and 75% percentile and median, whiskers indicate 1.5× inter‐quartile range. P values were generated using a paired Wilcoxon rank sum test. FIGURE S2: Changes in TRB parameters alongside cladribine therapy. (A) Number of TRB rearrangement templates. (B) Percent of baseline (M00) repertoire occupied by unique nucleotide TRB rearrangements divided into 12 quantiles (8.3% of unique TRB rearrangements per quantile) according to clonal rank. For month 12 and 24, only pre‐existing TRB clonotypes were quantified, identified by nucleotide overlap with the baseline sample. (C) Changes in TRB Simpson clonality correlated to flow cytometry‐derived change in percentages of CD8 terminally differentiated effector‐memory T cells (TEMRA) from baseline to 24‐month follow‐up. (D) Changes in unique nucleotide TRB rearrangements from baseline to month 12 and month 24, divided into patients with (n = 8 at M12 and n = 9 at M24) and without relapse activity (n = 10). FIGURE S3: Flow cytometry characterization of B‐cell subset frequencies. The plots depict percentages of B‐cell subsets quantified of CD19+ CD20+ B cells at baseline (M00) and 1 year after the 2nd cladribine cycle (M24) (MZ‐like = marginal zone‐like). Boxes indicate the 25% and 75% percentile and median, whiskers indicate 1.5× inter‐quartile range. P values were generated using a paired Wilcoxon rank sum test. [file ANA-99-1166-s002.docx]

**Supplementary materials**

**Supplementary Figures**

**Supplementary Figure 1**

**
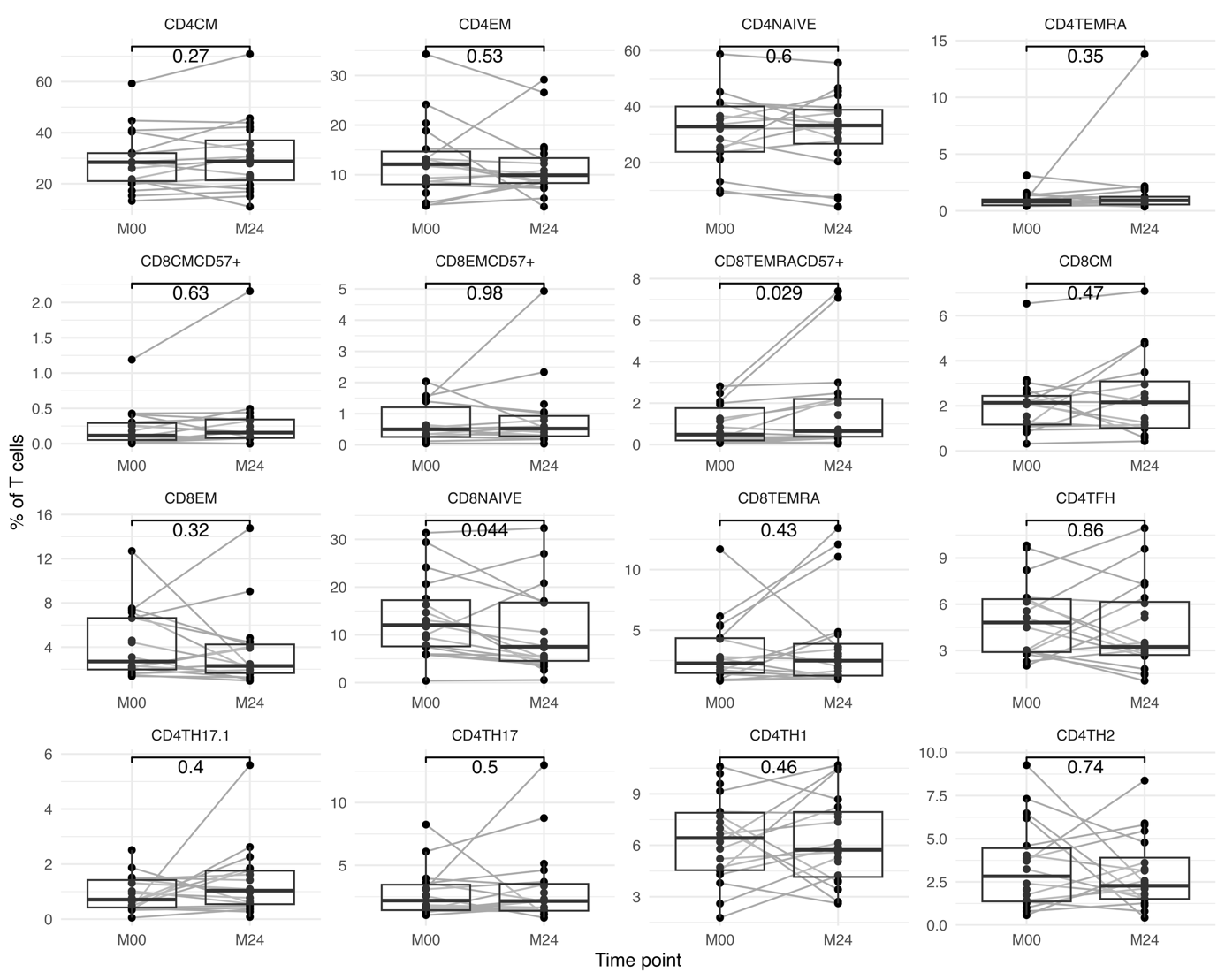
**

**Fig S1: Flow cytometry characterization of T-cell memory and T-helper subset frequencies**

The plots depict percentages of CD4 and CD8 memory T-cell subsets and CD4 T-helper cell (Th) subsets, quantified of CD3+ CD56- lymphocytes at baseline (M00) and one year after the 2^nd^ cladribine cycle (M24) (EM= effector-memory cells, CM=central-memory cells, TEMRA= terminally differentiated effector-memory cells, TH= T-helper cells, TFH= follicular T-helper cells). Boxes indicate the 25% and 75% percentile and median, whiskers indicate 1.5x inter-quartile range. P values were generated using a paired Wilcoxon rank sum test.

**Supplementary Figure 2**

**
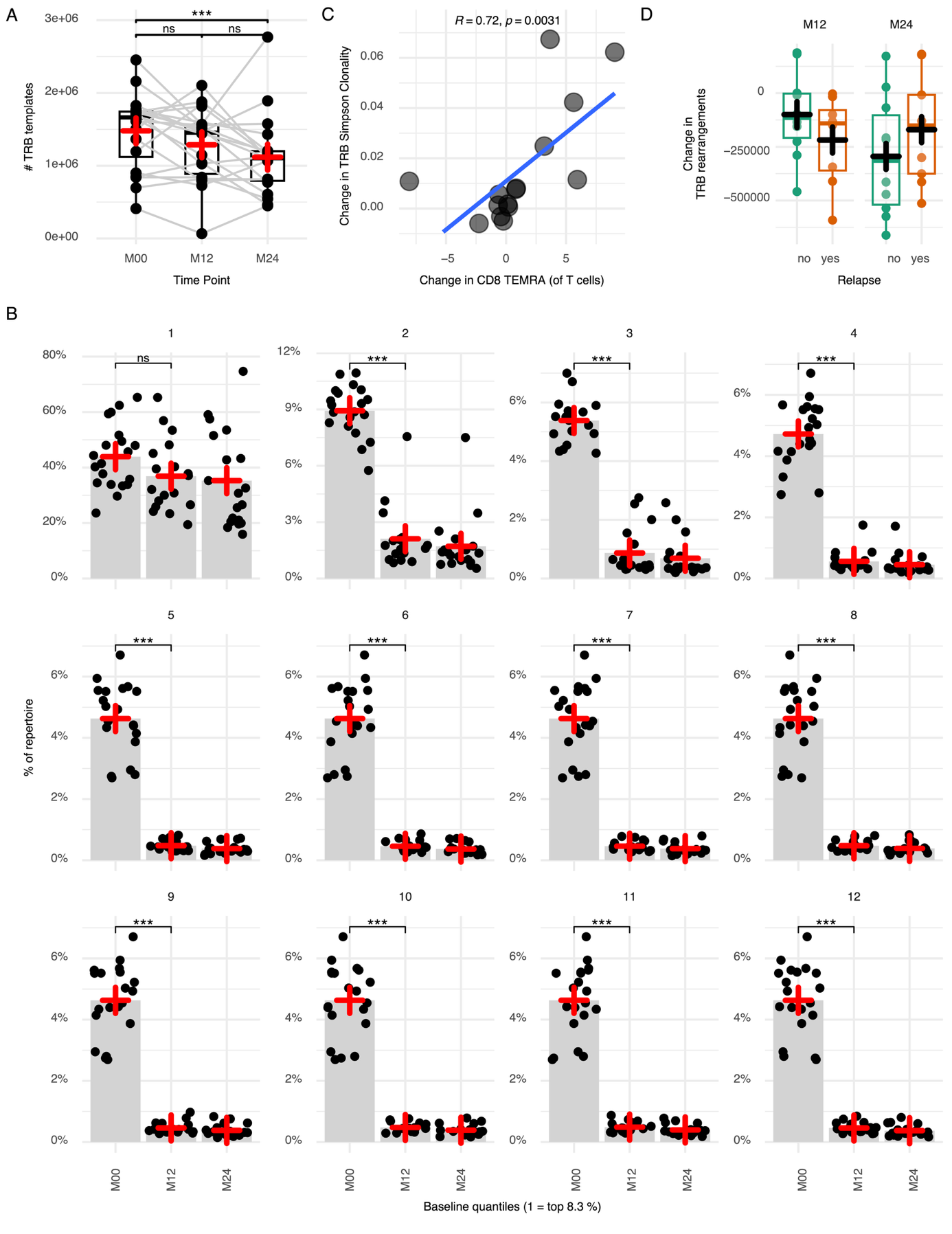
**

**Fig. S2: Changes in TRB parameters alongside cladribine therapy**

A: Number of TRB rearrangement templates.

B: Percent of baseline (M00) repertoire occupied by unique nucleotide TRB rearrangements divided into 12 quantiles (8.3% of unique TRB rearrangements per quantile) according to clonal rank. For month 12 and 24, only pre-existing TRB clonotypes were quantified, identified by nucleotide overlap with the baseline sample.

C: Changes in TRB Simpson clonality correlated to flow cytometry-derived change in percentages of CD8 terminally differentiated effector-memory T cells (TEMRA) from baseline to 24-month follow-up.

D: Changes in unique nucleotide TRB rearrangements from baseline to month 12 and month 24, divided into patients with (n=8 at M12 and n=9 at M24) and without relapse activity (n=10).

**Supplementary Figure 3**

**
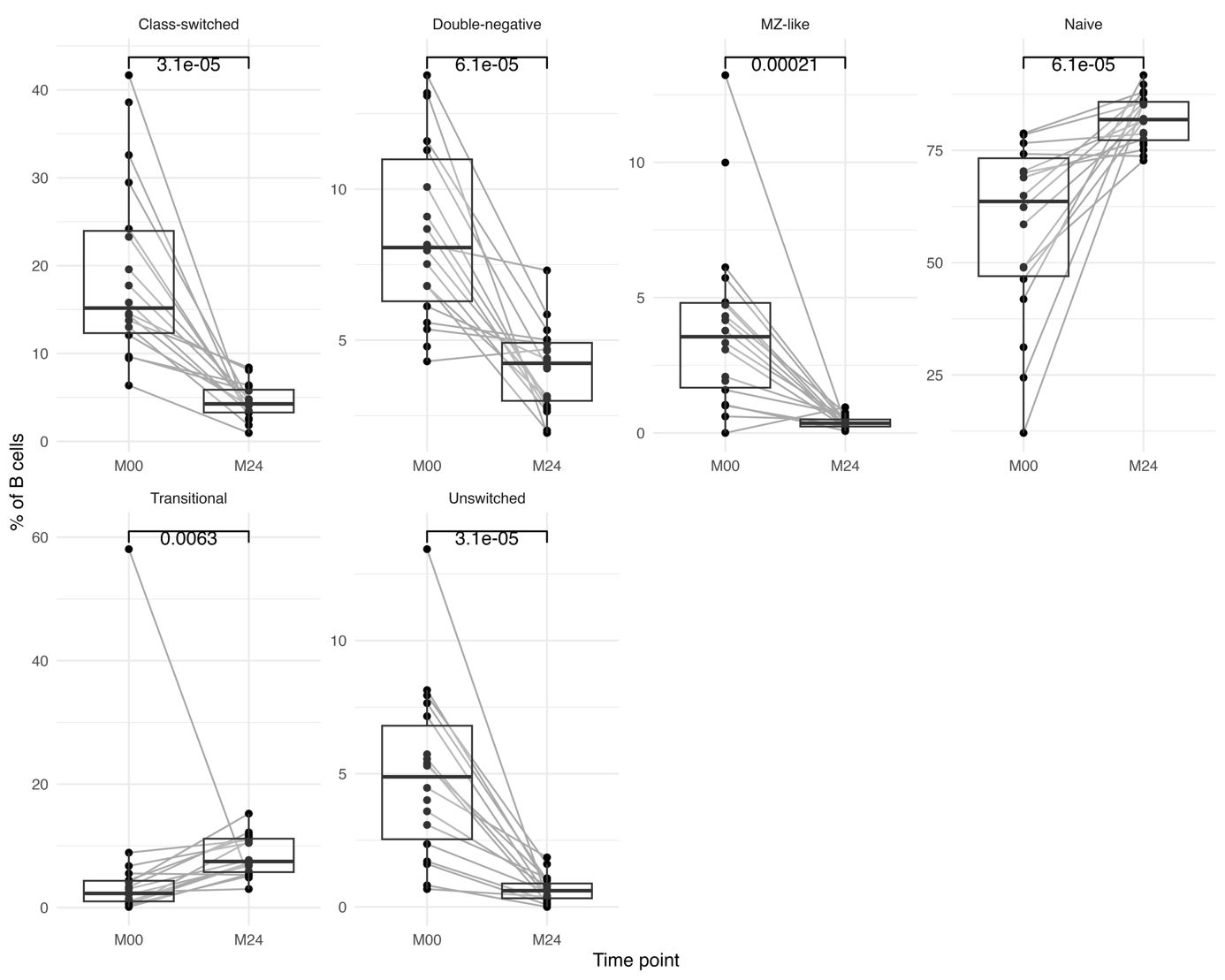
**

**Fig S3: Flow cytometry characterization of B-cell subset frequencies**

The plots depict percentages of B-cell subsets quantified of CD19+ CD20+ B cells at baseline (M00) and one year after the 2^nd^ cladribine cycle (M24) (MZ-like=marginal zone-like). Boxes indicate the 25% and 75% percentile and median, whiskers indicate 1.5x inter-quartile range. P values were generated using a paired Wilcoxon rank sum test.

**Supplementary Table 1: Correlations of immune receptor sequencing parameters with flow cytometry quantifications and list of antigen-specific TRB sequences**

Sheets 1-4 contain Spearman coefficients (column B) and p values (column C) from correlations of flow cytometry-derived changes (column A: tcell, th1 and bcell indicate the flow cytometry panel) in immune cell subset frequencies with changes in numbers of TRB clonotypes/rearrangements (sheet 1: Tcell_corr_clonotypes), TRB clonality (sheet 2: Tcell_corr_clonality), numbers of IGH clonotypes (sheet 3: Bcell_corr_clonotypes), and IGH mean SHM (sheet 4: Bcell_corr_shm). Sheet 5 (TRB_sequences_list) contains all antigen-specific TRB sequences extracted from public repositories and used for quantification of Fig. 1I, including organism (column A: EBV, CMV, Influenza A and SARS-CoV-2), protein (column B), epitope (column C), HLA restriction (column D), TRB variable gene restriction (column E), TRB junctional gene restriction (column F), and the TRB CDR3 amino acid sequence (column G).
